# Effects of Nd: YAG laser capsulotomy on central macular thickness: a systematic review and meta-analysis

**DOI:** 10.3389/fmed.2026.1891913

**Published:** 2026-07-21

**Authors:** Yipeng Deng, Yanling Lu, Ying Liu, Yongran Li

**Affiliations:** 1 Guangzhou Panyu Bright Eye Hospital, Guangzhou, China; 2 Center of Ophthalmology, Heyou Hospital, Foshan, China

**Keywords:** lasers, solid-state, macular thickness, meta-analysis, posterior capsule opacification, posterior capsulotomy

## Abstract

**Background:**

This study presents the first systematic review and meta-analysis evaluating central macular thickness (CMT) changes following Nd: YAG laser capsulotomy for posterior capsule opacification (PCO), providing evidence-based theoretical support for resolving controversies in postoperative macular edema.

**Methods:**

The study protocol was prospectively registered with PROSPERO (CRD420261390298). Systematic searches of PubMed, Embase, Web of Science, Scopus, Ovid Medline, and Cochrane Library were conducted from inception through May 1, 2026, following PRISMA and MOOSE guidelines. Studies were selected based on the PICOS framework: patients with posterior capsule opacification receiving Nd: YAG capsulotomy, comparing baseline and postoperative CMT. Exclusion criteria encompassed animal studies, secondary literature, non-English publications, and studies lacking CMT data. A total of 3 RCTs and 20 cohort studies met the eligibility criteria. Primary outcomes were CMT changes at postoperative day 1, week 1, month 1, and month 3. Risk of bias was assessed using Cochrane RoB 2.0 and Newcastle-Ottawa Scale. Meta-analyses were performed using Review Manager 5.4.1 and Stata 16.0, applying fixed effects or random-effects models based on heterogeneity (*I*^2^), with publication bias evaluated via Egger’s test and funnel plots. Additional analyses included meta-regression (ethnicity, study design, sample size, steroid use) and subgroup analyses (laser energy, capsulotomy size, OCT devices, baseline BCVA).

**Results:**

Analysis of 23 studies (1,762 eyes) demonstrated significant CMT increase at postoperative week 1 (MD: 8.72 μm; *p* < 0.001; *I*^2^ = 85%), month 1 (MD: 3.69 μm; *p* < 0.001; *I*^2^ = 12%), month 3 (MD: 5.07 μm; *p* < 0.001; *I*^2^ = 0%), with no change at day 1 (MD: 0.06 μm; *p* = 0.97; *I*^2^ = 0%). At week 1, greater CMT change occurred with laser energy ≥ 50 mJ and baseline BCVA ≥ 0.5 logMAR. Meta-regression showed no significant associations for ethnicity (*p* = 0.06), study design (*p* = 0.63), steroid use (*p* = 0.83), or sample size (*p* = 0.23). The overall risk of bias was considered moderate, while no significant publication bias was identified.

**Conclusion:**

This meta-analysis suggested a possible temporal pattern of CMT increases following Nd: YAG laser capsulotomy, with changes observed from week 1 through month 3 but not clearly at day 1. Subgroup analyses prompted by heterogeneity at week 1 indicated that higher energy levels and poorer baseline vision might be associated with greater thickness changes, while meta-regression did not show consistent associations with ethnicity, study design, steroid use, or sample size.

## 1 Background

Posterior capsule opacification (PCO), a prevalent complication following cataract surgery, demonstrates a cumulative incidence rate of 20.7% at 2 years postoperatively, increasing to 28.4% at 5 years (1, 2). This condition originates from aberrant proliferation of residual lens epithelial cells on the posterior capsule, forming opaque deposits that progressively impair visual acuity (3, 4). Extensive research has focused on preventive strategies, including the implementation of sharp-edged intraocular lenses (IOLs) and hydrophobic acrylic materials to mitigate PCO risk (5, 6). Neodymium-doped yttrium aluminum garnet (Nd: YAG) laser capsulotomy was the standard therapeutic intervention for PCO. This technique employs photodisruption to create an optical opening in the opacified posterior capsule, achieving success rates exceeding 95% in restoring functional visual acuity (7, 8). Nevertheless, despite its established efficacy, notable complications associated with this procedure warrant careful clinical consideration.

Nd: YAG laser capsulotomy carries risks of complications spanning both anterior and posterior segments, with commonly observed adverse effects including intraocular pressure (IOP) fluctuations, IOLs damage, and cystoid macular edema (9). Specifically regarding IOP elevation may occur through multiple mechanisms: liberated debris obstructing the trabecular meshwork, laser-induced trabeculitis from shockwave energy, neurovascular dysregulation, pupillary block phenomena, and inflammatory swelling of angle structures in predisposed eyes (10, 11). Concurrently, the procedure may displace intraocular implants, thereby altering the effective lens position and necessitating subsequent refractive correction for optimal visual rehabilitation. More critically, disruption of the blood-aqueous barrier facilitates vitreous turbulence and release of pro-inflammatory mediators, establishing conditions favorable for macular edema development. Among the most vision-threatening sequelae is cystoid macular edema (CME), wherein laser-triggered inflammation compromises the blood-retinal barrier, permitting fluid accumulation in the macular retina (12). This complication holds profound clinical significance since macular integrity fundamentally governs central visual function (13, 14).

Significant disagreement exists in the literature regarding Nd: YAG laser capsulotomy’s actual capacity to induce macular thickening or edema (15). While some optical coherence tomography (OCT) studies report measurable postoperative increases in central macular thickness suggesting subclinical edema, others found no significant macular changes following posterior capsulotomy (16). To our knowledge, no systematic review has comprehensively evaluated macular thickness alterations after this procedure. As the first systematic review and meta-analysis synthesizing global evidence on post Nd: YAG capsulotomy central macular thickness changes, this study aims to determine whether statistically significant thickness variations occur. Our findings will enhance postoperative macular monitoring vigilance among clinicians and provide evidence-based guidance for clinical management.

## 2 Methods

### 2.1 Search strategy for study selection

This systematic review adhered to the Preferred Reporting Items for Systematic Reviews and Meta-Analyses (PRISMA) guidelines and the Meta-analysis of Observational Studies in Epidemiology (MOOSE) checklist (Supplementary File S1 and Supplementary File S2) (17–19). The study protocol was prospectively registered with the International Prospective Register of Systematic Reviews (PROSPERO; Registration ID: CRD420261390298). Two independent investigators (DY and LYa) conducted a comprehensive search of six databases (PubMed, Embase, Web of Science, Scopus, Ovid Medline, and Cochrane Library) from inception through May 1, 2026. The search used a combination of Medical Subject Headings (MeSH) terms and free-text keywords: (“posterior capsulotomy” OR “lasers, solid-state” OR “neodymium-doped yttrium aluminum garnet” OR “Nd: YAG”) AND (“macular thickness” OR “foveal thickness”). Additional studies were identified through manual searches of reference lists, with full search strategies provided in the Supplementary File S3.

Eligibility criteria were defined according to the PICOS framework (20): Population—patients diagnosed with posterior capsule opacification; Intervention—Nd: YAG laser capsulotomy; Comparison—central macular thickness before and after Nd: YAG posterior capsulotomy; Outcome—changes in central macular thickness at postoperative day 1, week 1, month 1, and month 3; Study design—cohort studies or randomized controlled trials (RCTs) in English. Exclusion criteria included: (1) animal studies; (2) secondary literature (e.g., reviews, case reports, or letters); (3) studies lacking central macular thickness outcomes; (4) non-English publications; (5) studies with documentation errors in primary data were included solely for systematic review but excluded from meta-analysis.

Citavi v5.3 (Swiss Academic Software) was utilized for reference management and duplicate removal. The screening process was carried out in two stages by two independent reviewers (DY and LYa): (1) title and abstract screening; (2) full-text assessment against the inclusion and exclusion criteria. Discrepancies were resolved through discussion with a third investigator (LYo).

### 2.2 Data extraction

Data extraction was conducted using a predefined template that captured details such as the first author, publication year, country, ethnicity, gender, age, baseline laser BCVA, baseline laser IOP, capsule size, techniques, total energy, postoperative medication, OCT Devices and central macular thickness at postoperative day 1, week 1, month 1, and month 3. All included studies measured central macular thickness via OCT. Preoperative and postoperative measurements for all included eyes were extracted directly as reported in the original studies. Experienced operators performed multiple scans per eye, selecting the scan with the highest signal strength for inclusion. All definitions strictly followed standardized descriptions from the original studies. Two investigators (DY and LYa) independently performed blinded data extraction. Discrepancies were resolved through consensus with a third reviewer (LYo).

### 2.3 Risk of bias assessment

RCTs were evaluated using the Cochrane Risk of Bias 2.0 (RoB 2.0) checklist (22 items), which covers bias arising from the randomisation process, bias due to deviations from intended interventions, bias due to missing outcome data, bias in measurement of the outcome and bias in selection of the reported result (21). Each domain was assessed using three risk categories (low risk, some concerns, high risk), culminating in an overall risk of bias judgment for each outcome. The methodological quality of cohort studies was assessed using the Newcastle-Ottawa Scale (NOS), which evaluates three domains: participant selection, group comparability, and ascertainment of exposure, with a maximum score of 9 (22, 23). Two independent investigators (DY and LYa) conducted the risk-of-bias assessments, resolving discrepancies through discussion with a third reviewer (LYo). All RCTs were included, whereas cohort studies scoring < 5 on the NOS were classified as low quality with a high risk of bias and excluded from the meta-analysis to ensure robustness.

### 2.4 Statistical analysis

Statistical analyses were conducted using Review Manager (RevMan) v5.4.1 (Cochrane Collaboration), Comprehensive meta analysis (CMA) v3.0 (Biostat‌) and Stata v16.0 (Stata Corp). Comparisons encompassed changes in central macular thickness from preoperative baseline to postoperative day 1, week 1, month 1, and month 3. Continuous outcomes were expressed as mean ± standard deviation. Mean differences (MD) with 95% confidence intervals (CI) were calculated for outcomes with consistent units across studies. MD were calculated as postoperative value minus baseline value. Heterogeneity was assessed using *χ*^2^ tests and *I*^2^ statistics: fixed-effect models were applied when *p* > 0.10 and *I*^2^ < 50%; random-effects models were used otherwise. When significant heterogeneity was detected (*p* ≤ 0.10 or *I*^2^ ≥ 50%), we employed meta-regression and subgroup analyses to identify heterogeneity sources (24). Parameters reported completely across all included studies (ethnicity, document type, sample size, postoperative topical steroid) were analyzed using meta-regression. For parameters with incomplete reporting (total energy, capsule size, OCT devices, baseline laser BCVA), subgroup analyses were performed.

This dual approach ensured methodological appropriateness based on data availability. Sensitivity analyses (leave-one-out method) were performed for outcomes with ≥ 3 studies. As correlation coefficients were not reported in the primary studies, we performed sensitivity analyses using change means and standard deviations (SD) and different pre-post correlation (0.3, 0.5, and 0.7), with a random-effects model applied. Additionally, sensitivity analyses excluding bilateral-eye studies were conducted to evaluate potential bias. Publication bias was assessed using funnel plots and Egger’s test for all outcomes (25). Statistical significance was set at *p* < 0.05.

## 3 Results

### 3.1 Literature search results

**Figure 1:**
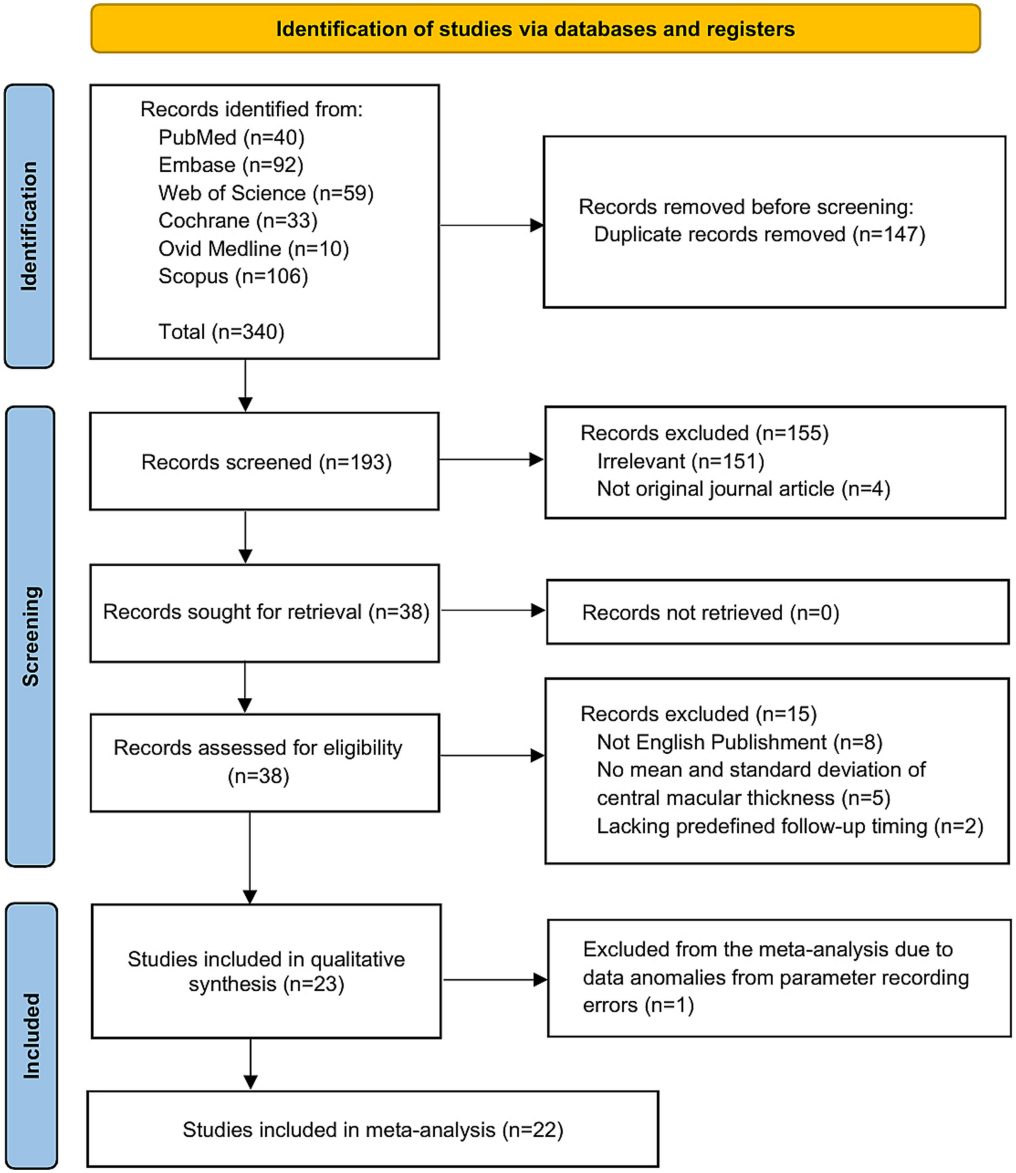
PRISMA Flowchart of selection of studies for inclusion in the meta-analysis.

The database search initially identified 340 articles. After removing 147 duplicates, titles, abstracts, and article types of the remaining 193 articles were screened. A total of 155 articles were excluded due to irrelevance to the topic ( = 151) or ineligible publication types (*n* = 4). Full-text assessments of the remaining 38 articles led to the exclusion of 8 non-English publications, 5 articles that did not report mean and standard deviation of central macular thickness, 2 articles lacking predefined follow-up timing. 1 article (26) was excluded due to anomalous data, with the preoperative CMT reported at an implausible value of approximately half the postoperative measurement, suggesting a documentation error. Consequently, 23 articles were included in the qualitative synthesis and 22 articles were included in meta-analysis. The selection process adhered to PRISMA standards, as illustrated in the flow diagram (Figure 1).

### 3.2 Primary outcomes and qualitative synthesis

This systematic review included 23 studies (26–48) (Table 1) published between 2010 and 2025, comprising 16 studies from Turkey (26, 27, 29, 32–34, 36, 40–48), 4 from India (28, 31, 35, 37), 2 from Egypt (30, 38) and 1 from Nepal (39), with participants of Caucasian, Asian and Arab. All included cases presented with PCO following unilateral or bilateral cataract surgery. The studies included 3 RCTs (28, 35, 37) and 20 cohort studies designs (26, 27, 29–34, 36, 38–48). The mean patient age ranged from 54 to 71.5 years in the 20 analyzed studies (27–30, 32–45, 47, 48), though age data were unreported in 3 studies (26, 31, 46). Sex distribution was reported in all 23 studies (26–48). Preoperative BCVA (logMAR) was reported in 16 studies (26, 27, 29–35, 39, 40, 44–48), with mean values ranging from 0.23 to 0.92. Intraocular pressure (IOP) data were documented in 19 studies (26, 27, 29–35, 37–41, 43–46, 48), showing mean values between 12 mmHg and 17.37 mmHg.

**Table 1:** Characteristics of included studies in meta-analysis.

Studies/year	Country	Ethnicity	Number of eyes	Sex (M/F)	Age (mean ± SD)	Baseline BCVA (logMAR) (mean ± SD)	Baseline IOP (mmHg) (mean ± SD)	Capsule size (mm) (mean ± SD)	Techniques	Total energy (mJ) (Mean ± SD)	Postoperative medication	OCT devices	Follow-up time
Atlihan et al. (27)	Turkey	Caucasian	34	12/22	63.46 ± 11.46	0.69 ± 0.33	14.47 ± 3.17	4.0–4.5	Circular	<50 mJ	Topical Brimonidine 0.15% bid + topical prednisolone acetate 1% five times daily for 1 week	DRI Triton SS-OCT (Topcon, Japan)	Week 1, month 1
Jha et al. (27)	India	Asian	48	18/30	59.69 ± 10.5	NA	NA	NA	NA	22.96 ± 4.98	Topical Nepafenac 0.1% for 1 week	Cirrus Model 500 SD-OCT (Carl Zeis, Germany)	Week 1, month 1, month 3
Küçük et al. (29)	Turkey	Caucasian	34	15/17	68.7 ± 8.6	0.47 ± 0.23	13.6 ± 2.6	NA	Cross	25.3 ± 7.3	Topical Fluorometholone 0.1% qid + topical brimonidine 0.15% bid for 1 week	DRI Triton SS-OCT (Topcon, Japan)	Week 1, month 1
Abokahla et al. (30)	Egypt	Arab	35	18/17	70 ± 3.6	0.4 ± 0.18	14.4 ± 1.5	NA	NA	NA	NA	RS-3000 SD-OCT (Nidek, Japan)	Month 1
Kaur et al. (31)	India	Asian	Group 1,175 group 2,175	Group 1 98/77 group 2 93/82	NA	Group 1 0.40 ± 0.15 group 2 0.49 ± 0.28	Group 1 14.50 ± 6.73 group 2 14.40 ± 5.46	3	NA	NA	Group 1 topical Loteprednol etabonate 0.5% qid for 1st week, tid for 2nd week, bid for 3rd week and qd for 4th week group 2 topical bromfenac 0.09% bid for 4 weeks	Spectralis SD-OCT (Heidelberg, Germany)	Week 1, month 1
Ciftci et al. (32)	Turkey	Caucasian	Group 1 56 group 2 61	Group 1 22/26 group 2 22/28	Group 1 69.8 ± 4.9 group 2 69.1 ± 9.9	Group 1 0.44 ± 0.36 group 2 0.48 ± 0.30	Group 1 15.9 ± 2.2 group 2 15.8 ± 2.4	4	NA	NA	Topical brinzolamide 1% + Timolol 0.5% combination drops bid and topical prednisolone acetate 1% qid for 1 week	RTVue XR SD-OCT (Optovue, America)	Month 1
Icoz et al. (34)	Turkey	Caucasian	32	17/13	60.1 ± 8.9	0.52 ± 0.21	13.5 ± 2.1	NA	NA	NA	Topical anti-glaucomatous + topical medium potency corticosteroid for 1 week	Cirrus SD-OCT (Carl Zeiss, America)	Week 1, month 1
Urfalioglu et al. (33)	Turkey	Caucasian	15	9/6	68.33 ± 5.23	0.42 ± 0.07	16.2 ± 1.47	4.0–4.5	NA	<50 mJ	Topical brimonidine 0.15% bid + topical prednisolone acetate 1% five times daily for 1 week	RTVue XR Avanti SD-OCT (Optovue, America)	Week 1, month 1
Jinagal et al. (35)	India	Asian	Group 1 36 group 2 30 group 3 24	33/57	58.38 ± 12.99	Group 1 0.50 ± 0.51 group 2 0.23 ± 0.43 group 3 0.38 ± 0.49	Group 1 15.28 ± 3.69 group 2 17.37 ± 3.18 group 3 14.25 ± 2.85	NA	NA	NA	Group 1 topical betamethasone 0.1% tid for 2 weeks group 2 topical nepafenac 0.1% tid for 2 weeks group 3 no postoperative medication	Spectralis SD-OCT (Heidelberg, America)	Month 1
Atilgan et al. (36)	Turkey	Caucasian	Group 1 25 group 2 25 group 3 25	Group 1 14/11 group 2 12/13 group 3 15/10	Group 1 63.78 ± 18.0 group 2 69.76 ± 11.2 group 3 65.75 ± 14.0	NA	NA	3–4	NA	Group 1 41.5 ± 23.6 group 2 51.7 ± 22.5 group 3 57.7 ± 29.7	Group 1 topical nepafenac 0.1% qid for 1 week group 2 topical fluorometholone 0.1% qid for 1 week group 3 no postoperative medication	Spectralis SD-OCT (Heidelberg, Germany)	Day 1, week 1, month 1
Deshwal et al. (27)	India	Asian	Group 1 75 group 2 75	Group 1 46/29 group 2 41/34	Group 1 58.97(range 54–65) group 2 61.11(range 57–67)	NA	Group 1 12.56 ± 1.63 group 2 12.4 ± 1.88	3	Cross	NA	Group 1 topical prednisolone acetate 1% qid for 1 week + topical bromfenac 0.09% bid for 6 weeks group 2 topical prednisolone acetate 1% qid for 1 week	Spectralis SD-OCT (Heidelberg, Germany)	Week 1
Vatansever et al. (26)	Turkey	Caucasian	41	18/17	NA	0.28 ± 0.13	13 ± 2.41	3–4	NA	NA	Topical prednisolone acetate 1% qid for 1 week	RS-3000 SD-OCT (Nidek, Japan)	Not included in meta-analysis
Alhagaa et al. (38)	Egypt	Arab	Group 1,100 group 2,100	Group 1 54/46 group 2 52/48	Group 1 61.1 ± 6.4 group 2 60.8 ± 5.9	NA	Group 1 15.4 ± 2.64 group 2 16.1 ± 2.36	Group 1 3.39 ± 0.29 group 2 4.71 ± 0.39	NA	NA	Topical prednisolone acetate 1% qid + topical timolol 0.5% bid + topical moxifloxacin qid for 5 days	Cirrus SD-OCT (Carl Zeiss, Germany)	Day 1, week 1, month 1, month 3
Parajuli et al. (39)	Nepal	Asian	Group 1 56 group 2 40	54/29	Group 1 62.72 ± 11.14 group 2 60.68 ± 14.70	Group 1 0.68 ± 0.36 group 2 0.69 ± 0.36	Group 1 14.51 ± 2.53 group 2 13.60 ± 2.78	NA	NA	Group 1 26.64 ± 12.92 group 2 81.96 ± 32.10	Topical dexamethasone 0.1% qid + topical ciprofloxacin 0.3% qid for 1 week	Cirrus SD-OCT (Carl Zeiss, America)	Month 1
Cevher et al. (40)	Turkey	Caucasian	42	19/23	63.7 ± 1.06	0.72 ± 0.55	13.6 ± 3.14	4.0–4.5	NA	69.75 ± 3.36	Topical prednisolone acetate 1% qid + topical brimonidine tartrate 0.2% bid for 1 week	SD-OCT/SLO (OPKO, America)	Week 1, month 1
Yilmaz and Yilmaz (41)	Turkey	Caucasian	42	20/22	66.8 ± 9.3	NA	15.6 ± 2.1	4.0	Cross or circular	44.85	Topical apraclonidine bid + topical dexamethasone five times daily for 5 days	Spectralis SD-OCT (Heidelberg, Germany)	Week 1, month 1, month 3
Yilmaz et al. (42)	Turkey	Caucasian	Group 1 22 group 2 20 group 3 22 group 4 24	48/40	66.2 ± 5.8	NA	NA	NA	Cross	Group 1 25.8 ± 7.9 group 2 27.5 ± 9.4 group 3 26.3 ± 9.8 group 4 26.0 ± 9.3	Group 1 and group 3 fluorometholon 0.1% qid for 1 week group 2 and Group 4 fluorometholon 0.1% qid + ketorolac 0.5% qid for 1 week	Cirrus SD-OCT (Carl Zeiss, America)	Day 1, week 1, month 1, month 3
Yuvacı et al. (43)	Turkey	Caucasian	28	13/15	63.17 ± 9.67	NA	14.25 ± 2.74	4	Cross or circular	41.93 ± 14.83	Topical prednisolone phosphate 0.5% qid for 2 weeks	Spectralis SD-OCT (Heidelberg, Germany)	Day 1, month 1, month 3
Kara et al. (44)	Turkey	Caucasian	Group 1 30 group 2 30	Group 1 10/19 group 2 7/21	Group 1 56.8 ± 19.2 group 2 57.3 ± 15.4	Group 1 0.40 ± 0.15 group 2 0.31 ± 0.16	Group 1 16.7 ± 2.9 group 2 15.6 ± 1.4	3–4	Group 1 Cross group 2 circular	Group 1 54.9 ± 19.0 group 2104.7 ± 37.3	Topical steroid for 1 week	NA	Day 1, week 1, month 1, month 3
Karahan et al. (45)	Turkey	Caucasian	Group 1 36 group 2 32	Group 1 16/20 group 2 17/15	Group 1 70.1 ± 7.8 group 2 71.5 ± 7.3	Group 1 0.61 ± 0.15 group 2 0.66 ± 0.18	Group 1 15.44 ± 2.73 group 2 15.37 ± 3.61	Group 1 3.43 ± 0.34 group 2 4.56 ± 0.47	NA	Group 1 57.6 ± 8.9 group 2 61.0 ± 12.7	Topical prednisolone acetate 1% qid + topical apraclonidine hydrochloride 0.5% bid for 5 days	NA	Week 1, month 1, month 3
Ari et al. (46)	Turkey	Caucasian	Group 1 16 group 2 14	19/11	NA	Group 1 0.68 ± 0.49 group 2 0.92 ± 0.44	Group 1 13.3 ± 3.6 group 2 12 ± 2.3	NA	NA	Group 1 58 ± 18 group 2,117 ± 36	Topical prednisolone acetate 1% qid + apraclonidine hydrochloride 0.5% bid for 3 days	Stratus 3.0 TD-OCT (Carl Zeiss, America)	Week 1, month 1, month 3
Kara et al. (47)	Turkey	Caucasian	33	14/19	54 ± 1	0.35 ± 0.19	NA	3–4	Cross	19.2 ± 7.6	Topical steroid for 1 week	Stratus 3.0 TD-OCT (Carl Zeiss, America)	Week 1, month 1, month 3
Altiparmak et al. (48)	Turkey	Caucasian	54	23/31	63.7 ± 15.9	0.47 ± 0.3	15.7 ± 2.4	NA	Cross	40.8 ± 23.9	Topical brimonidine tartrate 0.2% bid and topical prednisolone acetate 1% qid for 1 week	Stratus 3.0 TD-OCT (Carl Zeiss, America)	Day 1, week 1, month 1, month 3

BCVA best-corrected visual acuity, IOP intraocular pressure, OCT optical coherence tomography, SS-OCT swept-source optical coherence tomography, SD-OCT spectral-domain optical coherence tomography, TD-OCT time-domain optical coherence tomography, NA not applicable.

Among the 20 included studies (26–29, 31–33, 36–48) reporting YAG laser posterior capsulotomy parameters, documentation of capsule size, surgical techniques, and total energy varied in completeness. The mean capsule size ranged from 3 to 4.5 mm across all relevant studies. Regarding surgical techniques, 6 studies (29, 37, 42, 44, 47, 48) exclusively employed a cross technique, 2 utilized a circular technique (27, 44), and 2 reported using either approach (41, 43). The mean total energy delivered ranged from 19.2 to 117 mJ.

Postoperative medication regimens were reported in 22 studies (26–29, 31–48), though 3 failed to specify detailed therapeutic protocols (34, 44, 47) (including drug names, administration frequency, and treatment duration). The primary pharmacologic agents comprised topical corticosteroids (e.g., fluorometholone 0.1%, prednisolone acetate 1%, dexamethasone 0.1%), nonsteroidal anti-inflammatory drugs (NSAIDs; e.g., bromfenac 0.09%, nepafenac 0.1%), and intraocular pressure-lowering medications. Notably, several studies administered no postoperative medication. Regarding central macular thickness assessment, 22 studies (26–43, 45–48) documented the optical coherence tomography (OCT) devices employed, predominantly from Topcon, Carl Zeiss Meditec, Nidek, Heidelberg Engineering, Optovue, and OPKO/OTI. Comprehensive details are presented in Table 1.

### 3.3 Quality assessment

**Figure 2:**
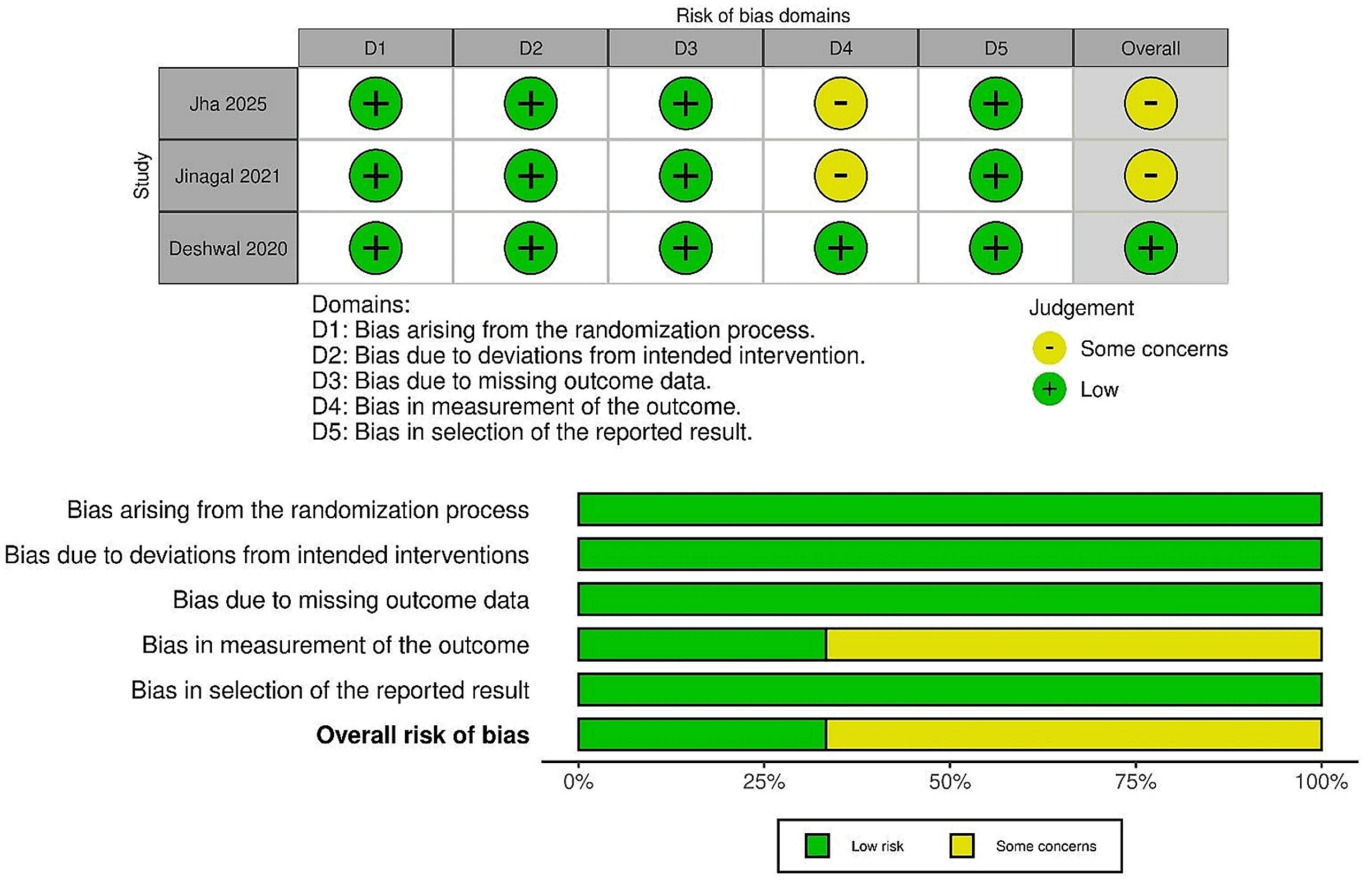
Risk of bias summary for randomized controlled trials.

Among all the included RCTs, all 3 RCTs (28, 35, 37) were judged to have low risk or some concerns across all domains. 2 RCTs had some concern concerning bias in measurement of the outcome (Figure 2). The cohort studies (27–30, 32–45, 47, 48) scored between 5 and 7 points on the NOS, demonstrating moderate to high quality (Table 2).

**Table 2:** Risk of bias assessment of included cohort studies.

Studies	Selection				Comparability	Outcome			Score
	1	2	3	4	5	6	7	8	
Atlihan et al. (27)	1	1	1	1	1	0	1	0	6
Küçük et al. (29)	1	1	1	1	1	0	1	0	6
Abokahla et al. (30)	1	1	1	1	1	0	1	1	7
Kaur et al. (31)	1	1	1	1	0	0	1	0	5
Ciftci et al. (32)	1	1	1	1	1	0	1	0	6
Icoz et al. (34)	1	1	1	1	1	0	1	0	5
Urfalioglu et al. (33)	1	1	1	1	1	0	1	0	6
Atilgan et al. (36)	1	1	1	1	1	0	1	0	6
Vatansever et al. (26)	1	1	1	1	1	1	1	0	7
Alhagaa et al. (38)	1	1	1	1	1	0	1	0	6
Parajuli et al. (39)	1	1	1	1	1	0	1	0	6
Cevher et al. (40)	1	1	1	1	1	0	1	0	6
Yilmaz and Yilmaz (41)	1	1	1	1	0	0	1	0	5
Yilmaz et al. (42)	1	1	1	1	0	0	1	0	5
Yuvacı et al. (43)	1	1	1	1	1	1	1	0	7
Kara et al. (44)	1	1	1	1	1	0	1	0	6
Karahan et al. (45)	1	1	1	1	1	0	1	0	6
Ari et al. (46)	1	1	1	1	0	0	1	0	5
Kara et al. (47)	1	1	1	1	0	0	1	0	5
Altiparmak et al. (48)	1	1	1	1	1	0	1	0	6

The Newcastle–Ottawa Scale for cohort studies, NOS: 1 = Representativeness of the exposed cohort; 2 = Selection of the non-exposed cohort; 3 = Ascertainment of exposure; 4 = Demonstration that outcome of interest was not present at start of study; 5 = Comparability of cohorts on the basis of the design or analysis controlled for confounders; 6 = Assessment of outcome; 7 = Was follow-up long enough for outcomes to occur; 8 = Adequacy of follow-up of cohorts.

Publication bias was evaluated using funnel plots across all 4 outcomes of meta-analyses. 3 outcomes were symmetrical scatter distribution alongside the axis, indicating an absence of publication bias. The remaining 1 outcome employing random-effects models, where auxiliary lines were not generated. Nevertheless, this study distributed evenly and symmetrically on both sides of the axis, suggesting an absence of publication bias (Supplementary File S4). Egger’s tests demonstrated nonsignificant publication bias for all four outcomes at specified timepoints: postoperative day 1 (*p* = 0.566), week 1 (*p* = 0.979), month 1 (*p* = 0.069), and month 3 (*p* = 0.381), with all *p*-values > 0.05 (Supplementary File S4). However, the ability to detect bias was limited for the day 1 analysis (8 studies), meaning its results should be interpreted with caution.

### 3.4 Results of meta-analysis

#### 3.4.1 Central macular thickness at baseline and postoperative day 1

**Figure 3:**
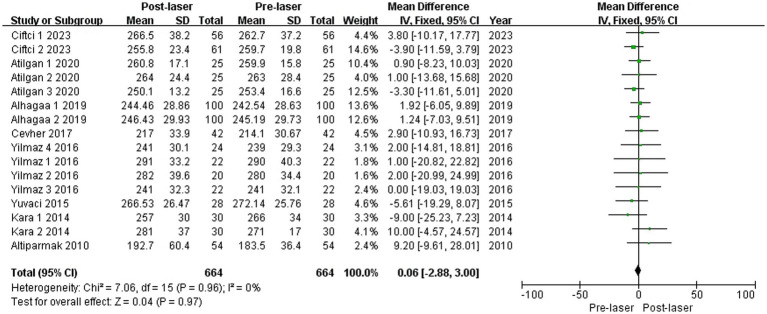
Forest plot of central macular thickness changes from baseline to postoperative day 1.

8 studies involving 664 eyes (Figure 3 and Table 3) showed no significant difference in central macular thickness at baseline and postoperative day 1 (MD = 0.06 μm, 95% CI: −2.88 to 3.00; *p* = 0.97), with nonsignificant heterogeneity (*I*^2^ = 0%, *p* = 0.96).

**Table 3:** Meta-analysis summary of central macular thickness changes from baseline to postoperative.

Parameters	Number of studies	Eyes	Pooled mean difference (μm)	95% CI	*P*-value	*I* ^2^
Day 1	8	664	0.06	[−2.88, 3.00]	0.97	0%
Week 1—overall	17	1,355	8.72	[3.90, 13.53]	<0.001^***^	85%
Total energy (mJ)						
≥ 50	/	250	8.79	[1.68, 15.89]	0.02^*^	56%
< 50	/	373	6.06	[−1.30, 13.43]	0.11	67%
Not given	/	732	11.87	[2.07, 21.67]	0.02^*^	95%
Capsule size (mm)						
≥ 4	/	265	14.27	[−5.45, 33.99]	0.16	91%
< 4	/	804	3.95	[−0.23, 8.13]	0.06	72%
Not given	/	286	11.89	[3.63, 20.16]	<0.01^**^	65%
OCT devices						
SS—OCT	/	68	0.31	[−19.25, 19.88]	0.97	43%
SD—OCT	/	1,042	8.74	[2.72, 14.76]	0.004^**^	89%
TD—OCT	/	117	12.74	[−0.25, 25.73]	0.05	68%
Not given	/	128	7.86	[−3.70, 19.43]	0.18	55%
Baseline laser BCVA (logMAR)						
≥ 0.5	/	206	13.86	[7.58, 20.13]	<0.001^***^	12%
< 0.5	/	546	1.82	[−0.70, 4.34]	0.16	0%
Not given	/	603	11.05	[2.05, 20.05]	0.02^*^	91%
Month 1	21	1,577	3.69	[2.16, 5.22]	<0.001^***^	12%
Month 3	10	651	5.07	[2.13, 8.00]	<0.001^***^	0%

^*^ indicates *P* < 0.05, ^**^ indicates *P* < 0.01, ^***^ indicates *P* < 0.001; OCT optical coherence tomography, SS—OCT swept-source optical coherence tomography, SD—OCT spectral-domain optical coherence tomography, TD—OCT time-domain optical coherence tomography, BCVA best-corrected visual acuity.

#### 3.4.2 Central macular thickness at baseline and postoperative week 1

**Figure 4:**
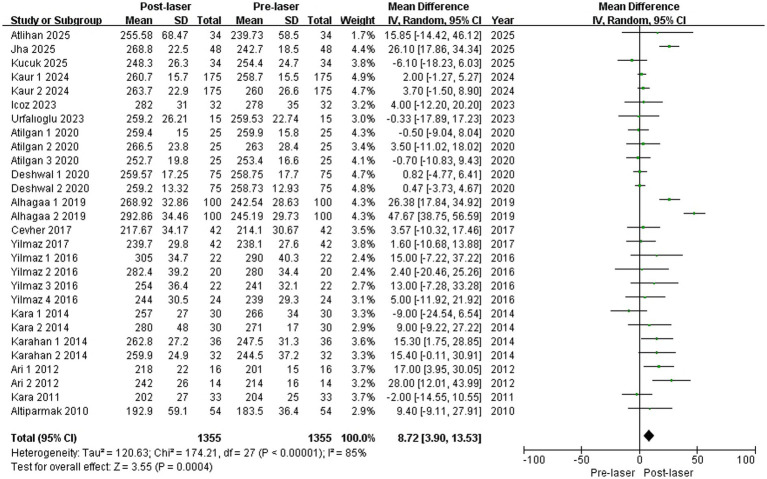
Forest plot of central macular thickness changes from baseline to postoperative week 1.

17 studies involving 1,355 eyes (Figure 4 and Table 3) showed significant difference in central macular thickness at baseline and postoperative week 1 (MD = 8.72 μm, 95% CI: 3.90 to 13.53; *p* < 0.001), with significant heterogeneity (*I*^2^ = 85%, *p* < 0.001).

Subgroup analyses (Table 3) revealed differential postoperative effects: higher energy (≥ 50 mJ) showed significant increase (MD: 8.79 μm, 95% CI: 1.68 to 15.89, *p* = 0.02, *I*^2^ = 56%) versus non-significant effects at lower energy (MD: 6.06 μm, 95% CI: −1.30 to 13.43, *p* = 0.11, *I*^2^ = 67%), while unreported energy studies exhibited strong effects (MD: 11.87 μm, 95% CI: 2.07 to 21.67, *p* = 0.02, *I*^2^ = 95%). For capsulotomy size, ≥ 4 mm showed non-significant increase (MD: 14.27 μm, 95% CI: −5.45 to 33.99, *p* = 0.16, *I*^2^ = 91%) compared to < 4 mm (MD: 3.95 μm, 95% CI: −0.23 to 8.13, *p* = 0.06, *I*^2^ = 72%), with unreported size studies being significant (MD: 11.89 μm, 95% CI: 3.63 to 20.16, *p* < 0.01, *I*^2^ = 65%). Analysis by OCT device type showed that only SD-OCT studies demonstrated a significant CMT increase (MD: 8.74 μm, 95% CI: 2.72 to 14.76, *p* = 0.004, *I*^2^ = 89%). No significant changes were observed in SS-OCT (MD: 0.31 μm, *p* = 0.97), TD-OCT (MD: 12.74 μm, *p* = 0.05), or studies with unreported device type (MD: 7.86 μm, *p* = 0.18). Notably, eyes with poor baseline vision (BCVA ≥ 0.5) had pronounced effects (MD: 13.86 μm, 95% CI: 7.58 to 20.13, *p* < 0.001, *I*^2^ = 12%), whereas better vision (BCVA < 0.5) showed minimal change (MD: 1.82 μm, 95% CI: −0.70 to 4.34, *p* = 0.16, *I*^2^ = 0%); unreported BCVA studies were significant (MD: 11.05 μm, 95% CI: 2.05 to 20.05, *p* = 0.02, *I*^2^ = 91%).

Univariate analysis (Supplementary File S6) revealed no significant predictors of central macular thickness change: ethnicity (Caucasian) (*p* = 0.06), RCT design (*p* = 0.63), no postoperative topical steroids (*p* = 0.83), or small sample size (< 40) (*p* = 0.23). Multivariate analysis confirmed no independent associations (all *p* > 0.05).

#### 3.4.3 Central macular thickness at baseline and postoperative month 1

**Figure 5:**
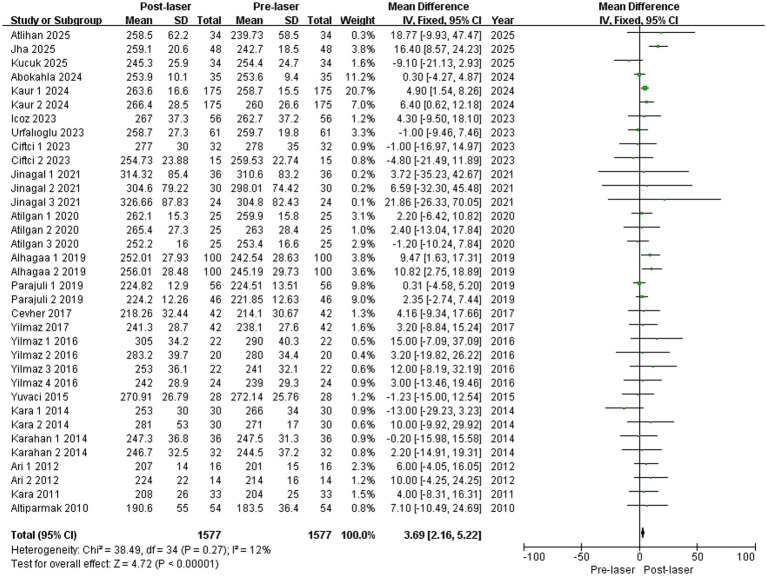
Forest plot of central macular thickness changes from baseline to postoperative month 1.

21 studies involving 1,577 eyes (Figure 5 and Table 3) showed significant difference in central macular thickness at baseline and postoperative month 1 (MD = 3.69 μm, 95% CI: 2.16 to 5.22; *p* < 0.001), with nonsignificant heterogeneity (*I*^2^ = 12%, *p* = 0.27).

#### 3.4.4 Central macular thickness at baseline and postoperative month 3

**Figure 6:**
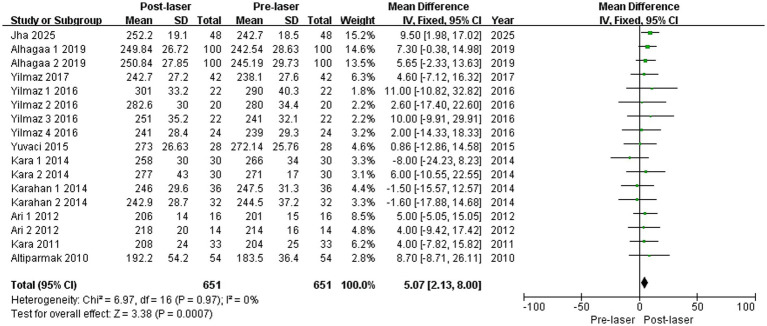
Forest plot of central macular thickness changes from baseline to postoperative month 3.

10 studies involving 651 eyes (Figure 6 and Table 3) showed significant difference in central macular thickness at baseline and postoperative month 3 (MD = 5.07 μm, 95% CI: 2.13 to 8.00; *p* < 0.001), with nonsignificant heterogeneity (*I*^2^ = 0%, *p* = 0.97).

#### 3.4.5 Sensitivity analyses

Sensitivity analyses were conducted to evaluate the robustness of the findings. Leave-one-out analyses demonstrated consistent results: for week 1, month 1, and month 3 outcomes, the effect direction remained unchanged with preserved statistical significance (*p* < 0.05), while the day 1 analysis showed no alteration in effect direction or significance level (*p* > 0.05) (Supplementary File S5). Additionally, sensitivity analyses excluding bilateral-eye studies yielded results consistent with the primary analyses across all four time points (Supplementary File S5). Furthermore, sensitivity analyses using change means and SDs and different pre-post correlation coefficients (0.3, 0.5, and 0.7) for paired data handling showed that the pooled estimates remained stable and consistent with the primary results across all assumed correlation values, indicating potential robustness of the findings (Supplementary File S5).

## 4 Discussion

This systematic review and meta-analysis represented the first comprehensive evaluation of central macular thickness (CMT) changes following Nd: YAG laser capsulotomy for posterior capsule opacification (PCO). Our analysis of 23 studies encompassing 1,762 eyes demonstrates a clear temporal pattern of CMT alterations. No significant change was observed at postoperative day 1, while statistically significant increases were detected at week 1, month 1, and month 3. These findings provide crucial evidence-based guidance for postoperative monitoring protocols, suggesting that the macular changes may begin at the first postoperative week. The clinical relevance of these findings stems from their ability to advance our understanding of the debated relationship between Nd: YAG laser capsulotomy and macular changes. While the procedure remains the preferred treatment for PCO management due to its effectiveness and convenience, concerns about posterior segment complications have persisted. Our quantitative synthesis confirms that measurable CMT changes do occur, though the degree of these changes suggests they represent mild inflammation rather than significant cystoid macular edema.

The observed CMT alterations may be explained by several interconnected biological mechanisms. Available evidence suggests that laser-induced intraocular inflammation is likely a contributing factor to macular thickening (49). The laser process generates pressure waves that disrupt the blood-retinal barrier, leading to increased inflammatory substance release and subsequent blood vessel leakage (50). This inflammatory process promotes fluid accumulation in the macular retina, showing as measurable thickness increases on optical coherence tomography (OCT). Although the primary target is the clouded posterior capsule, it is plausible that the laser’s effects can influence nearby eye structures through energy spread and inflammatory substance diffusion (51, 52).

Emerging evidence also points to possible involvement of the choroid in this inflammatory response. Research indicates that inflammation caused by laser treatment affects the choroidal blood vessels and contributes to changes in choroidal vascular measurements (27, 29). Inflammatory processes and the release of signaling molecules can affect choroidal vessels, leading to widening and filling of the choroidal blood network. This choroidal involvement might help explain the persistence of CMT changes observed at month 3, as choroidal inflammation can have longer-lasting effects than front-of-the-eye inflammation. Optical coherence tomography angiography (OCTA) has provided additional insights into microvascular changes following Nd: YAG capsulotomy. This technology quantifies blood flow and vessel density, often showing reduced vascular perfusion in both superficial and deep retinal layers (53). These microvascular alterations may represent the earliest sign of laser-induced inflammation, potentially preceding measurable CMT increases (54). Studies note transient foveal avascular zone changes that typically normalize within 1 month, suggesting that microvascular responses are more dynamic than structural thickness alterations (27).

Our subgroup analyses at postoperative week 1, conducted to investigate potential sources of heterogeneity, revealed several important factors influencing the CMT response to Nd: YAG capsulotomy. Laser energy emerged as a significant factor, with higher energy settings (≥50 mJ) producing more noticeable CMT increases (MD = 8.79 μm) compared to lower energy applications (MD = 6.06 μm). This dose–response relationship may support the involvement of mechanical and inflammatory mechanisms in laser-induced macular changes, as higher energy levels could contribute to greater pressure forces and more extensive tissue disturbance.

Baseline visual acuity also proved to be an important determinant of CMT response at postoperative week 1. Patients with poorer preoperative vision (BCVA ≥0.5 logMAR) exhibited substantially greater CMT increases (MD = 13.86 μm) compared to those with better baseline sight (MD = 1.82 μm). This finding may reflect several underlying factors: patients with worse vision may have more severe PCO requiring more intensive laser treatment with higher energy levels and longer duration, potentially inducing stronger inflammatory responses; longer disease duration allowing for chronic inflammatory changes; or pre-existing retinal vulnerability.

Subgroup analysis by OCT device type revealed differential CMT changes at postoperative week 1, with only SD-OCT studies showing a statistically significant increase (MD: 8.74 μm, *p* = 0.004). The non-significant results in the smaller SS-OCT and TD-OCT subgroups should be interpreted cautiously due to limited study numbers. It should be noted, however, that previous research has suggested SD-OCT may provide slightly superior resolution in detecting subtle macular thickness or morphological changes compared to TD-OCT and SS-OCT, though these devices still demonstrate good consistency overall (55–57). While all measurements were performed using the same device before and after surgery in each study, factors such as posterior capsule opacification may have affected baseline measurement resolution, potentially influencing results. Additionally, inherent variability in machine repeated measurements could also contribute to observed differences (58, 59).

Capsulotomy size showed a tendency toward larger CMT increases with bigger openings (≥ 4 mm: MD = 14.27 μm vs. < 4 mm: MD = 3.95 μm) at postoperative week 1, though this difference did not reach statistical significance (*p* = 0.16), possibly due to limited sample size in the larger capsulotomy group. Larger openings may allow greater movement of inflammatory substances into the vitreous cavity, potentially explaining this trend. Studies have reported that the vitreous can shift toward the front chamber due to membrane rupture during laser treatment. Such vitreous movement can lead to changes in the retina (60).

Meta-regression analyses were performed to further explore heterogeneity sources, examining ethnicity, study design, sample size, and postoperative topical steroid use. However, none of these factors demonstrated statistically significant associations with CMT changes (all *p* > 0.05), suggesting that the observed heterogeneity may be attributable to other unmeasured variables or the inherent clinical diversity across studies. Additionally, the generalizability of the findings may be limited by the geographical concentration of the included studies, predominantly from Turkey, which may have also limited the statistical power and interpretability of the meta-regression analysis for ethnicity.

The demonstrated CMT increases following Nd: YAG capsulotomy highlight the importance of appropriate anti-inflammatory prevention. Medication is advisable after Nd: YAG laser capsulotomy, as current evidence supports the effectiveness of both topical NSAIDs and corticosteroids in reducing postoperative inflammation (35, 61, 62). Topical NSAIDs may be preferred in certain situations, particularly when steroid use could be problematic, such as in patients who respond poorly to steroids, those with recurrent herpes eye infections, and individuals with higher risk for cystoid macular edema—notably diabetic patients, who exhibit increased susceptibility due to functional impairment of retinal capillaries that may intensify inflammatory responses to laser treatment (63–66). Some evidence suggests that macular thickness in untreated patients may show transient increases during the initial postoperative period but often returns toward baseline levels within 1 month, indicating that the inflammatory response may be self-limiting in many cases; nevertheless, prophylactic treatment could be considered for individuals with elevated risk factors. Regarding intraocular pressure management, routine preventive medications are generally unnecessary, as postoperative pressure elevations are typically minimal and clinically insignificant (61).

This suggests that the pooled estimate at this time point should be interpreted with caution, as the underlying sources of between-study variability remain incompletely explained. Second, the predominance of observational studies introduces potential risks of selection bias and residual confounding. Additionally, the restriction to English-language publications, which resulted in the exclusion of eight non-English articles, may have introduced language bias. Additionally, the inclusion of studies in this meta-analysis that incorporated data from both eyes of the same patient may have introduced confounding factors. To enhance the reliability of future syntheses, it is advisable to prioritize studies that report outcomes at the patient level or include data from only one eye per participant.

However, several limitations of this meta-analysis should be acknowledged. First, substantial heterogeneity was observed in the week-1 analysis (*I*^2^ = 85%), which persisted despite subgroup and meta-regression analyses. In addition, the statistically significant yet relatively modest increases in central macular thickness after Nd: YAG capsulotomy should be interpreted with caution regarding clinical relevance, particularly for the week 1 results which showed considerable heterogeneity. The observed changes were approximately 9 μm at week 1, 4 μm at month 1, and 5 μm at month 3. These subtle changes may approach the range of typical measurement variability associated with optical coherence tomography and could reflect mild subclinical responses rather than clinically meaningful edema. These subtle changes may approach the range of typical measurement variability associated with optical coherence tomography and could reflect mild subclinical responses rather than clinically meaningful edema. Another limitation is that our analysis could not fully account for several patient and treatment characteristics that might influence CMT changes, including diabetes status, diabetic retinopathy, baseline CMT, the time between cataract surgery and the laser procedure, and the type of lens implant. The original studies did not report or adjust for these variables in a consistent way, and these unmeasured factors likely contributed to the observed heterogeneity. Future studies should strive to systematically collect and adjust for these potential confounders to enhance the validity of findings (67–69).

The relatively brief follow-up period in many included studies restricts conclusions about long-term effects. Longer follow-up research is needed to understand the long-term course of changes after Nd: YAG laser treatment (70). Additionally, differences in OCT technology across studies may influence thickness measurements. Studies have found that image quality with older generation OCT systems could be affected by PCO, while imaging with newer spectral-domain OCT is rarely affected and generally provides reliable results. The progressive adoption of advanced OCT in recent studies likely improved measurement accuracy (71, 72).

This meta-analysis suggests several clinical implications: structured postoperative monitoring appears beneficial, particularly during the first week when CMT changes become detectable; high-energy laser settings and poorer baseline vision may warrant closer observation or extended anti-inflammatory prophylaxis; and although CMT increases are statistically significant, they are generally mild and infrequently progress to clinical cystoid macular edema. Future studies should focus on comparing anti-inflammatory regimens, assessing long-term outcomes of mild CMT changes, utilizing advanced imaging for microvascular evaluation, and personalizing laser parameters based on individual risk profiles through larger, multi-center collaborations. In summary, evidence indicates that Nd: YAG capsulotomy results in statistically significant CMT elevations detectable at week 1, month 1, and month 3 postoperatively, highlighting the essential role of vigilant monitoring in balancing procedural safety for PCO patients.

## Data Availability

The original contributions presented in the study are included in the article/Supplementary material, further inquiries can be directed to the corresponding author.

## References

[1] Schaumberg DA Dana MR Christen WG Glynn RJ. A systematic overview of the incidence of posterior capsule opacification. Ophthalmology. (1998) 105:1213–21. doi: 10.1016/s0161-6420(98)97023-3, 9663224

[2] Bhargava R Kumar P Phogat H Chaudhary KP. Neodymium-yttrium aluminium garnet laser capsulotomy energy levels for posterior capsule opacification. J Ophthalmic Vis Res. (2015) 10:37–42. doi: 10.4103/2008-322X.156101, 26005551 PMC4424716

[3] Belda JI Dabán JP Elvira JC O’Boyle D Puig X Pérez-Vives C . Nd:YAG capsulotomy incidence associated with five different single-piece monofocal intraocular lenses: a 3-year Spanish real-world evidence study of 8293 eyes. Eye (Lond). (2022) 36:2205–10. doi: 10.1038/s41433-021-01828-z, 34764439 PMC9581982

[4] Vasavada AR Praveen MR. Posterior capsule opacification after phacoemulsification: annual review. 10.1097/APO.0000000000000080. (2014) 3:235–40. doi: 10.1097/APO.0000000000000080 26107764

[5] Findl O Buehl W Bauer P Sycha T. Interventions for preventing posterior capsule opacification. Cochrane Database Syst Rev. (2007) 3:CD003738. doi: 10.1002/14651858.CD003738.pub2 17636731

[6] Maedel S Evans JR Harrer-Seely A Findl O. Intraocular lens optic edge design for the prevention of posterior capsule opacification after cataract surgery. Cochrane Database Syst Rev. (2021) 8:CD012516. doi: 10.1002/14651858.CD012516.pub2 34398965 PMC8406949

[7] Lighthizer N Johnson S Holthaus J Holthaus K Cherian B Swindell R . Nd:YAG laser capsulotomy: efficacy and outcomes performed by optometrists. Optom Vis Sci. (2023) 100:665–9. doi: 10.1097/OPX.0000000000002057, 37594749 PMC10662619

[8] de Senne FMB Temporini ER Arieta CEL Pacheco KD. Perception of difficulties with vision-related activities of daily living among patients undergoing unilateral posterior capsulotomy. Clinics. (2010) 65:459–68. doi: 10.1590/s1807-59322010000500002, 20535363 PMC2882539

[9] Grzybowski A Kanclerz P. Does Nd:YAG capsulotomy increase the risk of retinal detachment? Asia-Pacific journal of ophthalmology (Philadelphia, Pa). (2018) 7:339–44. doi: 10.22608/APO.2018275 30043556

[10] Gupta T Kashyap R Ganjoo S. Comparison of Bimatoprost 0.03% and Brimonidine 0.2% in managing intraocular pressure elevation following Nd: YAG laser capsulotomy for posterior capsule opacification. J Pharm Bioallied Sci. (2024) 16:S3142–5. doi: 10.4103/jpbs.jpbs_605_24, 39927002 PMC11805283

[11] Song Z Chen Z Li C Li Y Liu Y Lu P. Study of the relationship between the severity of posterior capsular opacification detected by objective detection techniques and visual acuity. J Cataract Refract Surg. (2024) 50:1020–5. doi: 10.1097/j.jcrs.0000000000001494, 38783488

[12] Wielders LHP Schouten JSAG Winkens B van den Biggelaar FJHM Veldhuizen CA Findl O . European multicenter trial of the prevention of cystoid macular edema after cataract surgery in nondiabetics: ESCRS PREMED study report 1. J Cataract Refract Surg. (2018) 44:429–39. doi: 10.1016/j.jcrs.2018.01.029 29778106

[13] Gill G Taylor Gonzalez D Sanghvi H Djulbegovic M Suleiman A Gupta S. Artificial intelligence for posterior capsule opacification. Front Med. (2025) 12:1695525. doi: 10.3389/fmed.2025.1695525, 41458487 PMC12741075

[14] Khalaff AG Emara B. A case report on acute cystoid macular edema days after YAG laser capsulotomy. Int J Surg Case Rep. (2025) 136:111960. doi: 10.1016/j.ijscr.2025.111960, 40987220 PMC12491868

[15] Borkenstein AF Borkenstein E-M. Neodymium-doped yttrium aluminum garnet (Nd: YAG) laser treatment in ophthalmology: a review of the most common procedures capsulotomy and iridotomy. Lasers Med Sci. (2024) 39:167. doi: 10.1007/s10103-024-04118-8, 38954050

[16] Alimanović Halilović E. Correlation between eye aperture diameter and complications in the posterior eye segment after Nd-YAG capsulotomy. Bosn J Basic Med Sci. (2008) 8:106–9. doi: 10.17305/bjbms.2008.2961, 18498257 PMC5698337

[17] Page MJ McKenzie JE Bossuyt PM . The PRISMA 2020 statement: an updated guideline for reporting systematic reviews. BMJ (Clinical research ed.). (2021) 372:n71. doi: 10.1136/bmj.n71 PMC8005924 33782057

[18] Stroup DF Berlin JA Morton SC Olkin I Williamson GD Rennie D . Meta-analysis of observational studies in epidemiology: a proposal for reporting. Meta-analysis of observational studies in epidemiology (MOOSE) group. JAMA. (2000) 283:2008–12. doi: 10.1001/jama.283.15.2008 10789670

[19] Atkins D Fink K Slutsky J Agency for Healthcare Research and Quality; North American Evidence-based Practice Centers. Better information for better health care: the Evidence-based Practice Center program and the Agency for Healthcare Research and Quality. Ann Intern Med. (2005) 142:1035–41. doi: 10.7326/0003-4819-142-12_part_2-200506211-00002 15968027

[20] Schardt C Adams MB Owens T Keitz S Fontelo P. Utilization of the PICO framework to improve searching PubMed for clinical questions. BMC Med Inform Decis Mak. (2007) 7:16. doi: 10.1186/1472-6947-7-16, 17573961 PMC1904193

[21] Sterne JAC Savović J Page MJ Elbers RG Blencowe NS Boutron I . RoB 2: a revised tool for assessing risk of bias in randomised trials. BMJ (Clinical research ed). (2019) 366:l4898. doi: 10.1136/bmj.l4898 31462531

[22] Lo CK-L Mertz D Loeb M. Newcastle-Ottawa scale: comparing reviewers' to authors' assessments. BMC Med Res Methodol. (2014) 14:45. doi: 10.1186/1471-2288-14-45, 24690082 PMC4021422

[23] Stang A. Critical evaluation of the Newcastle-Ottawa scale for the assessment of the quality of nonrandomized studies in meta-analyses. Eur J Epidemiol. (2010) 25:603–5. doi: 10.1007/s10654-010-9491-z, 20652370

[24] Higgins JPT Thompson SG. Quantifying heterogeneity in a meta-analysis. Stat Med. (2002) 21:1539–58. doi: 10.1002/sim.1186, 12111919

[25] Egger M Davey Smith G Schneider M Smith GD Minder C. Bias in meta-analysis detected by a simple, graphical test. BMJ. (1997) 315:629–34. doi: 10.1136/bmj.315.7109.629, 9310563 PMC2127453

[26] Vatansever M Dinç E Dursun Ö Adıgüzel U Yılmaz A Temel GÖ. The role of optical coherence tomography signal strength in the diagnosis and follow-up of patients with posterior capsular opacification treated with Nd:YAG laser capsulotomy. Turkish J Ophthalmol. (2020) 50:80378. doi: 10.4274/tjo.galenos.2019.80378, 32166941 PMC7086094

[27] Atlıhan YS Erkan Pota Ç Çetinkaya Yaprak A. Changes in macular microvasculature after Nd:YAG laser capsulotomy: an OCT-A study. Int Ophthalmol. (2025) 45:391. doi: 10.1007/s10792-025-03751-8, 41014396

[28] Jha R Chander A Suman Rastogi P Adlakha B. Effect of topical nepafenac versus fluorometholone on macular thickness after Nd:YAG capsulotomy. Indian J Clin Exp Ophthalmol. (2025) 11:551–7. doi: 10.18231/j.ijceo.v.11.i.3.30

[29] Küçük E Yeşilyurt H Çakmak M Zor KR Karataş MÇ. Evaluation of the effects of ND: YAG laser capsulotomy on choroidal thickness and choroidal vascularity index. Lasers Med Sci. (2025) 40:120. doi: 10.1007/s10103-025-04379-x, 40014149 PMC11868316

[30] Abokahla OA Ghoneim EM Mohamed MM. Assessment of retinal microvascular alterations after YAG laser capsulotomy using optical coherence tomography angiography. African Journal of Biological Sciences (South Africa). (2024) 6:5573–83. doi: 10.5281/zenodo.13356045

[31] Kaur S Bhartiya S Shukla P Sinha Y Singh N. To compare the effect of topical 0.5% loteprednol etabonate versus 0.09% bromfenac on macular thickness following Nd: yag laser capsulotomy in a tertiary care hospital. Int J Pharm Clin Res. (2024) 16:300–4.

[32] Ciftci F Filik A. Effects of Nd: YAG laser capsulotomy on visual acuity, central macular thickness, intraocular pressure and refraction in patients with diabetes mellitus. Med Rec. (2023) 5:249–54. doi: 10.37990/medr.1178529

[33] Urfalıoglu S Özdemir G Güler M Daghan B Öz F. The evaluation of the effect of Nd-YAG capsulotomy on posterior ocular vascular structures by optical coherence tomography angiography. Photodiagn Photodyn Ther. (2023) 42:103323. doi: 10.1016/j.pdpdt.2023.103323, 36773755

[34] Icoz M Tarım B Icoz SGG. The effect of Nd:YAG laser applied in the posterior capsule opacification on retinal and choroidal structures. Photodiagn Photodyn Ther. (2023) 43:103653. doi: 10.1016/j.pdpdt.2023.103653, 37295662

[35] Jinagal J Sahu S Gupta G Khurana S Gupta R Gupta PC . Quantification of inflammation following Nd:YAG laser capsulotomy and assessing the anti-inflammatory effects of Nepafenac 0.1% and betamethasone 0.1. Ocul Immunol Inflamm. (2021) 29:411–6. doi: 10.1080/09273948.2019.1668025, 31638843

[36] Atilgan CU Kosekahya P Yetkin E Caglayan M Goker YS Sendul SY. Effects of topical nepafenac and fluorometholone on macular thickness after posterior capsulotomy using neodymium-doped yttrium-aluminum-garnet laser. Beyoglu Eye J. (2020) 5:64–72. doi: 10.14744/bej.2020.96158, 35098066 PMC8784478

[37] Deshwal M Sethi HS Naik MP Gupta VS. Topical steroid alone vs a combination with a posterior segment NSAID after Nd-YAG capsulotomy: is the posterior segment NSAID really necessary? J Family Med Prim Care. (2020) 9:664–8. doi: 10.4103/jfmpc.jfmpc_461_19, 32318400 PMC7113968

[38] Alhagaa A Badawi N. Influence of yttrium aluminum garnet laser posterior capsulotomy opening size on intraocular pressure and central macular thickness. Delta J Ophthalmol. (2019) 20:157. doi: 10.4103/djo.djo_6_19

[39] Parajuli A Joshi P Subedi P Pradhan C. Effect of Nd:YAG laser posterior capsulotomy on intraocular pressure, refraction, anterior chamber depth, and macular thickness. Clin Ophthalmol. (2019) 13:945–52. doi: 10.2147/OPTH.S203677 31239636 PMC6559220

[40] Cevher S Koçluk Y Çetinkaya S Ünal F Çubuk M. Short-term effect of nd:yag laser capsulotomy on refraction, central macular thickness and retinal nerve fber layer thickness. Rev Bras Oftalmol. (2017) 76:186–9. doi: 10.5935/0034-7280.20170037

[41] Yilmaz T Yilmaz A. Long-term changes in subfoveal choroidal thickness and central macula thickness after Nd:YAG laser capsulotomy. Int Ophthalmol. (2017) 37:1003–8. doi: 10.1007/s10792-016-0353-x, 27699606

[42] Yılmaz U Küçük E Ulusoy DM Özköse A Ataş M Demircan S . The assessment of changes in macular thickness in diabetic and non-diabetic patients: the effect of topical ketorolac on macular thickness change after ND:YAG laser capsulotomy. Cutan Ocul Toxicol. (2016) 35:58–61. doi: 10.3109/15569527.2015.1017579 25799211

[43] Yuvacı İ Pangal E Yüce Y Yuvacı S Bayram N Ulusoy DM . Optic coherence tomography measurement of choroidal and retinal thicknesses after uncomplicated YAG laser capsulotomy. Arq Bras Oftalmol. (2015) 78:344–7. doi: 10.5935/0004-2749.20150091 26677034

[44] Kara N Evcimen Y Kirik F Agachan A Yigit FU. Comparison of two laser capsulotomy techniques: cruciate versus circular. Semin Ophthalmol. (2014) 29:151–5. doi: 10.3109/08820538.2013.874467, 24475914

[45] Karahan E Tuncer I Zengin MO. The effect of ND:YAG laser posterior capsulotomy size on refraction, intraocular pressure, and macular thickness. J Ophthalmol. (2014) 2024:846385. doi: 10.1155/2014/846385 PMC3958711 24724016

[46] Ari S Cingü AK Sahin A Çinar Y Çaça I. The effects of Nd:YAG laser posterior capsulotomy on macular thickness, intraocular pressure, and visual acuity. Ophthalmic Surg Lasers Imaging Retina. (2012) 43:395–400. doi: 10.3928/15428877-20120705-03, 22785102

[47] Kara N Yazici AT Bozkurt E Yildirim Y Demirok A Yilmaz OF. Which procedure has more effect on macular thickness: primary posterior continuous capsulorhexis (PPCC) combined with phacoemulsification or Nd:YAG laser capsulotomy? Int Ophthalmol. (2011) 31:303–7. doi: 10.1007/s10792-011-9461-9, 21842401

[48] Altiparmak UE Ersoz I Hazirolan D Koklu B Kasim R Duman S. The impact of Nd:YAG capsulotomy on foveal thickness measurement by optical coherence tomography. Ophthalmic Surg Lasers Imaging Retina. (2010) 41:67–71. doi: 10.3928/15428877-20091230-12 20143510

[49] Aslam TM Devlin H Dhillon B. Use of Nd:YAG laser capsulotomy. Surv Ophthalmol. (2003) 48:594–612. doi: 10.1016/j.survophthal.2003.08.002 14609706

[50] Xu H Chen M Forrester JV Lois N. Cataract surgery induces retinal pro-inflammatory gene expression and protein secretion. Invest Ophthalmol Vis Sci. (2011) 52:249–55. doi: 10.1167/iovs.10-6001 20720227

[51] Brézin AP Labbe A Schweitzer C Lignereux F Rozot P Goguillot M . Incidence of Nd:YAG laser capsulotomy following cataract surgery: a population-based nation-wide study—FreYAG1 study. BMC Ophthalmol. (2023) 23:417. doi: 10.1186/s12886-023-03134-6, 37845645 PMC10578013

[52] Ceylan A Aydin FO Karapapak M Aydın S Ozal SA Yildirim Y. Nd:YAG laser capsulotomy vs needle aspiration in intumescent cataracts: comparative study of complications and outcomes. J Cataract Refract Surg. (2024) 50:1123–7. doi: 10.1097/j.jcrs.0000000000001517, 38958988

[53] Little K Llorián-Salvador M Tang M Du X O'Shaughnessy Ó McIlwaine G . A two-stage laser-induced mouse model of subretinal fibrosis secondary to choroidal neovascularization. Transl Vis Sci Technol. (2020) 9:3. doi: 10.1167/tvst.9.4.3, 32818091 PMC7396176

[54] Özdamar Erol Y Güngör A Şekeryapan GB. Peripapillary and macular choroidal vascularity index in eyes with Fuchs' uveitis. Ocul Immunol Inflamm. (2022) 30:1853–8. doi: 10.1080/09273948.2021.1964031, 34410884

[55] Yeu E Berdahl JP Gupta PK Patterson M. Sensitivity and specificity of SS-OCT for detecting macular pathologies vs SD-OCT. J Cataract Refract Surg. (2024) 50:481–5. doi: 10.1097/j.jcrs.0000000000001394, 38192061 PMC11045393

[56] González-García AO Vizzeri G Bowd C Medeiros FA Zangwill LM Weinreb RN. Reproducibility of RTVue retinal nerve fiber layer thickness and optic disc measurements and agreement with stratus optical coherence tomography measurements. Am J Ophthalmol. (2009) 147:1067–74. doi: 10.1016/j.ajo.2008.12.032 19268891 PMC3465966

[57] Pierro L Zampedri E Milani P Gagliardi M Isola V Pece A. Spectral domain OCT versus time domain OCT in the evaluation of macular features related to wet age-related macular degeneration. Clinical ophthalmology (Auckland, NZ). (2012) 6:219–23. doi: 10.2147/OPTH.S27656, 22347793 PMC3280103

[58] Lee KM Lee EJ Kim T-W Kim H. Comparison of the abilities of SD-OCT and SS-OCT in evaluating the thickness of the macular inner retinal layer for Glaucoma diagnosis. PLoS One. (2016) 11:e0147964. doi: 10.1371/journal.pone.0147964, 26812064 PMC4727815

[59] Vizzeri G Weinreb RN Gonzalez-Garcia AO Bowd C Medeiros FA Sample PA . Agreement between spectral-domain and time-domain OCT for measuring RNFL thickness. Br J Ophthalmol. (2009) 93:775–81. doi: 10.1136/bjo.2008.150698, 19304586 PMC3465953

[60] Stark WJ Worthen D Holladay JT Murray G. Neodymium: YAG lasers. An FDA report. Ophthalmology. (1985) 92:209–12. doi: 10.1016/s0161-6420(85)34051-4 3982799

[61] Unal M Yücel I Akar Y. Brinzolamide 1% versus apraclonidine 0.5% to prevent intraocular pressure elevation after neodymium:YAG laser posterior capsulotomy. J Cataract Refract Surg. (2006) 32:1499–502. doi: 10.1016/j.jcrs.2006.04.014 16931262

[62] Mian OT Asif H Sandhu U Multani K Farooq AV Ding K . Noninfectious outcomes of intravitreal antibiotic steroid injection and topical nonsteroidal Antiinflammatory drugs versus triple drop therapy after cataract surgery. Am J Ophthalmol. (2024) 260:37–48. doi: 10.1016/j.ajo.2023.11.003, 37944685

[63] Endo N Kato S Haruyama K Shoji M Kitano S. Efficacy of bromfenac sodium ophthalmic solution in preventing cystoid macular oedema after cataract surgery in patients with diabetes. Acta Ophthalmol. (2010) 88:896–900. doi: 10.1111/j.1755-3768.2009.01582.x 19725815

[64] Song SH Baek SK Lee MW Lee YH. Effect of 0.1% Bromfenac for preventing macular edema after cataract surgery in patients with diabetes. Korean J Ophthalmol. (2020) 34:46–55. doi: 10.3341/kjo.2019.0044, 32037749 PMC7010466

[65] Hoffman RS Braga-Mele R Donaldson K Emerick G Henderson B Kahook M . Cataract surgery and nonsteroidal antiinflammatory drugs. J Cataract Refract Surg. (2016) 42:1368–79. doi: 10.1016/j.jcrs.2016.06.006, 27697257 PMC5531861

[66] Shorstein NH Liu L Waxman MD Herrinton LJ. Comparative effectiveness of three prophylactic strategies to prevent clinical macular edema after phacoemulsification surgery. Ophthalmology. (2015) 122:2450–6. doi: 10.1016/j.ophtha.2015.08.024, 26409728 PMC4658259

[67] Yotsukura E Torii H Saiki M Negishi K Tsubota K. Effect of neodymium:YAG laser capsulotomy on visual function in patients with posterior capsule opacification and good visual acuity. J Cataract Refract Surg. (2016) 42:399–404. doi: 10.1016/j.jcrs.2015.11.042, 27063520

[68] Wang H Li Y Wong MOM Chan CKM Yuen HKL Liu Z. Incidence and associated factors of Nd:YAG posterior capsulotomy after premium IOL implantation surgery: a systematic review and meta-analysis. Int J Surg. (2026) 112:4938–64. doi: 10.1097/js9.0000000000003855, 41556197

[69] Liu H Liu X Chen Y Wang D Li Y Chen H . Effect of Nd:YAG laser capsulotomy on the risk for retinal detachment after cataract surgery: systematic review and meta-analysis. J Cataract Refract Surg. (2022) 48:238–44. doi: 10.1097/j.jcrs.0000000000000755, 34538778

[70] Cagini C Pietrolucci F Lupidi M Messina M Piccinelli F Fiore T. Influence of pseudophakic lens capsule opacification on spectral domain and time domain optical coherence tomography image quality. Curr Eye Res. (2015) 40:579–84. doi: 10.3109/02713683.2014.941069, 25110908

[71] Tao S Liang F Fan S Wang M Zhang Y Liu X . Objective quantification of posterior capsule opacification using swept-source AS-OCT. J Cataract Refract Surg. (2025) 51:3–8. doi: 10.1097/j.jcrs.0000000000001546, 39254371

[72] Zhou Y Xiang J Xu F Jiang Z Liu F. Objective quantification of posterior capsule opacification after cataract surgery with swept-source optical coherence tomography. BMC Ophthalmol. (2023) 23:299. doi: 10.1186/s12886-023-03064-3, 37407917 PMC10324270

