# Supplementary material for: Effects of Nd: YAG laser capsulotomy on central macular thickness: a systematic review and meta-analysis
Source: Front Med (Lausanne). 2026 Jul 21;13:1891913. doi: 10.3389/fmed.2026.1891913 (PMC13434851; doi:10.3389/fmed.2026.1891913)
Supplement: Supplementary file 3 [file Supplementary_File_3.DOCX]

**Supplementary File 3**

| **PubMed** From inception to May 1st, 2026 | | |
| --- | --- | --- |
| #1 | ("posterior capsulotomy"[MeSH Terms] OR "lasers, solid-state"[MeSH Terms] OR "posterior capsulotomy"[Title/Abstract] OR "neodymium-doped yttrium aluminum garnet"[Title/Abstract] OR "Nd:YAG"[Title/Abstract]) AND ("macular thickness"[Title/Abstract] OR "foveal thickness"[Title/Abstract]) | **40** |
| **Web of science** From inception to May 1st, 2026 | | |
| #1 | ((((((AB=(posterior capsulotomy)) OR TI=(posterior capsulotomy)) OR AB=(neodymium-doped yttrium aluminum garnet)) OR TI=(neodymium-doped yttrium aluminum garnet)) OR AB=(Nd:YAG)) OR TI=(Nd:YAG)) | **36210** |
| #2 | ((((AB=(macular thickness)) OR TI=(macular thickness)) OR AB=(foveal thickness)) OR TI=(foveal thickness)) | **18038** |
| #3 | #1 AND #2 | **59** |
| **Cochrane** From inception to May 1st, 2026 | | |
| #1 | MeSH descriptor: [posterior capsulotomy] explode all trees | **20** |
| #2 | MeSH descriptor: [lasers, solid-state] explode all trees | **1286** |
| #3 | (posterior capsulotomy):ti,ab,kw | **279** |
| #4 | (neodymium-doped yttrium aluminum garnet):ti,ab,kw | **137** |
| #5 | (Nd YAG):ti,ab,kw | **1360** |
| #6 | #1 or #2 or #3 or #4 or #5 | **2401** |
| #7 | (macular thickness):ti,ab,kw | **3756** |
| #8 | (foveal thickness):ti,ab,kw | **813** |
| #9 | #7 or #8 | **3898** |
| #10 | #6 and #9 | **33** |
| **Embase** From inception to May 1st, 2026 | | |
| #1 | 'posterior capsulotomy'/exp | **5909** |
| #2 | 'lasers, solid-state '/exp | **34078** |
| #3 | 'posterior capsulotomy':ab,ti | **1023** |
| #4 | 'neodymium-doped yttrium aluminum garnet':ab,ti | **633** |
| #5 | 'Nd:YAG':ab,ti | **12380** |
| #6 | #1 OR #2 OR #3 OR #4 OR #5 | **42045** |
| #6 | 'macular thickness':ab,ti | **7584** |
| #7 | 'foveal thickness':ab,ti | **3019** |
| #8 | #7 OR #8 | **10230** |
| #9 | #6 AND #8 | **92** |
| **Medline** From inception to May 1st, 2026 | | |
| #1 | Tl posterior capsulotomy OR AB posterior capsulotomy OR Tl neodymium-doped yttrium aluminum garnet OR AB neodymium-doped yttrium aluminum garnet OR Tl Nd:YAG OR AB Nd:YAG | **1754** |
| #2 | Tl macular thickness OR AB macular thickness OR TI foveal thickness OR AB foveal thickness | **1560** |
| #3 | S1 OR S2 | **10** |
| **Scopus** From inception to May 1st, 2026 | | |
| #1 | (TITLE-ABS-KEY(posterior capsulotomy) OR TITLE-ABS-KEY(neodymium-doped yttrium aluminum garnet) OR TITLE-ABS-KEY(Nd YAG)) AND (TITLE-ABS-KEY(macular thickness) OR TITLE-ABS-KEY(foveal thickness)) | **106** |
