# Supplementary material for: Effects of Nd: YAG laser capsulotomy on central macular thickness: a systematic review and meta-analysis
Source: Front Med (Lausanne). 2026 Jul 21;13:1891913. doi: 10.3389/fmed.2026.1891913 (PMC13434851; doi:10.3389/fmed.2026.1891913)
Supplement: Supplementary file 4 [file Supplementary_File_4.DOCX]

**Supplementary File 4**

Figure 1: Funnel plot of central macular thickness changes from baseline to postoperative day 1

Figure 2: Egger’s test of central macular thickness changes from baseline to postoperative day 1

Figure 3: Funnel plot of central macular thickness changes from baseline to postoperative week 1

Figure 4: Egger’s test of central macular thickness changes from baseline to postoperative week 1

Figure 5: Funnel plot of central macular thickness changes from baseline to postoperative month 1

Figure 6: Egger’s test of central macular thickness changes from baseline to postoperative month 1

Figure 7: Funnel plot of central macular thickness changes from baseline to postoperative month 3

Figure 8: Egger’s test of central macular thickness changes from baseline to postoperative month 3
