# Supplementary material for: Effects of Nd: YAG laser capsulotomy on central macular thickness: a systematic review and meta-analysis
Source: Front Med (Lausanne). 2026 Jul 21;13:1891913. doi: 10.3389/fmed.2026.1891913 (PMC13434851; doi:10.3389/fmed.2026.1891913)
Supplement: Supplementary file 5 [file Supplementary_File_5.DOCX]

**Supplementary File 5**Figure 1: Sensitivity analysis of central macular thickness changes from baseline to postoperative day 1

Figure 2: Sensitivity analysis of central macular thickness changes from baseline to postoperative week 1

Figure 3: Sensitivity analysis of central macular thickness changes from baseline to postoperative month 1

Figure 4: Sensitivity analysis of central macular thickness changes from baseline to postoperative month 3

Figure 5: Sensitivity analysis of central macular thickness changes from baseline to postoperative day 1 with a presumed pre‑post correlation coefficient of 0.3

Figure 6: Sensitivity analysis of central macular thickness changes from baseline to postoperative day 1 with a presumed pre‑post correlation coefficient of 0.5

Figure 7: Sensitivity analysis of central macular thickness changes from baseline to postoperative day 1 with a presumed pre‑post correlation coefficient of 0.7

Figure 8: Sensitivity analysis of central macular thickness changes from baseline to postoperative week 1 with a presumed pre-post correlation coefficient of 0.3

Figure 9: Sensitivity analysis of central macular thickness changes from baseline to postoperative week 1 with a presumed pre-post correlation coefficient of 0.5

Figure 10: Sensitivity analysis of central macular thickness changes from baseline to postoperative week 1 with a presumed pre-post correlation coefficient of 0.7

Figure 11: Sensitivity analysis of central macular thickness changes from baseline to postoperative month 1 with a presumed pre-post correlation coefficient of 0.3

Figure 12: Sensitivity analysis of central macular thickness changes from baseline to postoperative month 1 with a presumed pre-post correlation coefficient of 0.5

Figure 13: Sensitivity analysis of central macular thickness changes from baseline to postoperative month 1 with a presumed pre-post correlation coefficient of 0.7

Figure 14: Sensitivity analysis of central macular thickness changes from baseline to postoperative month 3 with a presumed pre-post correlation coefficient of 0.3

Figure 15: Sensitivity analysis of central macular thickness changes from baseline to postoperative month 3 with a presumed pre-post correlation coefficient of 0.5

Figure 16: Sensitivity analysis of central macular thickness changes from baseline to postoperative month 3 with a presumed pre-post correlation coefficient of 0.7

Figure 17: Sensitivity analysis of central macular thickness changes from baseline to postoperative day 1 after excluding bilateral-eye studies

Figure 18: Sensitivity analysis of central macular thickness changes from baseline to postoperative week 1 after excluding bilateral-eye studies

Figure 19: Sensitivity analysis of central macular thickness changes from baseline to postoperative month 1 after excluding bilateral-eye studies

Figure 20: Sensitivity analysis of central macular thickness changes from baseline to postoperative month 3 after excluding bilateral-eye studies
