# Supplementary material for: Effects of Nd: YAG laser capsulotomy on central macular thickness: a systematic review and meta-analysis
Source: Front Med (Lausanne). 2026 Jul 21;13:1891913. doi: 10.3389/fmed.2026.1891913 (PMC13434851; doi:10.3389/fmed.2026.1891913)
Supplement: Supplementary file 6 [file Supplementary_File_6.DOCX]

**Supplementary File 6**

Table 1 Univariate meta-regression analyses of central macular thickness changes from baseline to postoperative week 1

|  | Number of eyes | β | 95%CI | P-value |
| --- | --- | --- | --- | --- |
| Ethnicity - Caucasian | 607 | -0.35 | [-0.71, 0.02] | 0.06 |
| RCTs | 198 | 0.13 | [-0.42, 0.69] | 0.63 |
| No postoperative topical steroid | 273 | 0.06 | [-0.46, 0.57] | 0.83 |
| Sample size < 40 | 469 | -0.22 | [-0.57, 0.14] | 0.23 |

Note: *indicates P < 0.05, **indicates P < 0.01, ***indicates P < 0.001.

Table 2 Multivariate meta-regression analyses of central macular thickness changes from baseline to postoperative week 1

|  | Number of eyes | β | 95%CI | P-value |
| --- | --- | --- | --- | --- |
| Ethnicity - Caucasian | 607 | -0.53 | [-1.22, 0.16] | 0.13 |
| RCTs | 198 | -0.21 | [-0.88, 0.45] | 0.52 |
| No postoperative topical steroid | 273 | -0.06 | [-0.58, 0.16] | 0.82 |
| Sample size < 40 | 469 | 0.10 | [-0.48, 0.68] | 0.73 |

Note: *indicates P < 0.05, **indicates P < 0.01, ***indicates P < 0.001.
